# Taurodeoxycholic, taurocholic, and glycocholic acids promote hepatic gluconeogenesis via TGR5 in dairy cows

**DOI:** 10.1186/s40104-025-01275-w

**Published:** 2025-10-30

**Authors:** Miaomiao Zhu, Yining Zheng, Shiyang Lou, Ruixu Zhang, Dingping Feng, Xinjian Lei, Lei Chen, Jianguo Wang, Junhu Yao, Lu Deng

**Affiliations:** 1 College of Animal Science and Technology, Northwest A&F University, Yangling, Shaanxi 712100 China; 2 College of Veterinary Medicine, Northwest A&F University, Yangling, Shaanxi 712100 China; 3 Shenzhen Research Institute, Northwest A&F University, Shenzhen, Guangdong 518000 China

**Keywords:** cAMP-response element binding protein, Gluconeogenesis, Glycocholic acid, Takeda G protein-coupled receptor 5, Taurocholic acid, Taurodeoxycholic acid

## Abstract

**Background:**

Ruminants and monogastric animals exhibit significant differences in gluconeogenic efficiency. In dairy cows, hepatic gluconeogenesis serves as the primary source of glucose. Metabolites modulate gluconeogenesis efficiency through allosteric regulation, redox state, and signal transduction pathways. However, the liver-enriched metabolites that regulate hepatic gluconeogenesis in dairy cows and their specific regulatory mechanisms remain incompletely characterized.

**Results:**

Six Holstein dairy cows and six Duroc × (Landrace × Yorkshire) (DLY) crossbred pigs served as research subjects. Employing non-targeted and targeted metabolomics, we discovered that three bile acids—taurodeoxycholic acid (TDCA), taurocholic acid (TCA), and glycocholic acid (GCA)—were highly enriched in Holstein dairy cows’ livers. In bovine hepatocytes, individual or combined stimulation of these bile acids significantly upregulated the expression of gluconeogenesis genes (*FBP1*, *PCK1* and *G6PC*) and enhanced glucose production. In fasting mice with induced gluconeogenesis, TDCA, TCA, and GCA increased fasting blood glucose levels, and pyruvate tolerance tests further revealed their capacity to enhance hepatic gluconeogenesis, enabling more efficient glucose synthesis from pyruvate. Mechanistically, these bile acids activated Takeda G protein-coupled receptor 5 (TGR5), elevated intracellular cAMP levels, and ultimately enhanced gluconeogenesis via the transcription factor cAMP-response element binding protein (CREB). Notably, a TGR5 inhibitor abrogated the stimulatory effects of TDCA, TCA, and GCA on hepatic gluconeogenesis in fasting mice.

**Conclusion:**

TDCA, TCA, and GCA are key metabolites promoting hepatic gluconeogenesis in dairy cows, with TGR5 as the pivotal receptor and the cAMP/PKA/CREB pathway as the critical downstream mechanism.

**Supplementary Information:**

The online version contains supplementary material available at 10.1186/s40104-025-01275-w.

## Introduction

For ruminants, the mode of glucose generation within their bodies differs from that of monogastric animals. The glucose is primarily produced via rumen fermentation and hepatic gluconeogenesis rather than through gastrointestinal digestion and absorption [1]. Notably, approximately 80% of the glucose required for energy maintenance and lactose synthesis in dairy cows is derived from hepatic gluconeogenesis [2]. In recent years, several studies have explored valuable metabolites by analyzing metabolic differences across species. For example, one study employed integrated metabolomics to systematically quantify and compare metabolite, lipid, and protein profiles across five mammalian milk sources: human, pig, cow, yak, and buffalo. The analysis revealed distinct compositional signatures among species, with significant interspecies variation in bioactive components [3]. Furthermore, considering the lower susceptibility of pigs to type 2 diabetes mellitus (T2DM), a comparative analysis of bile acid composition was conducted across humans, mice, and pigs. This study identified hyocholic acid species as a key component significantly enriched in swine. Mechanistic investigations further revealed that this bile acid enhanced glucose homeostasis, thereby mitigating the risk of T2DM development [4]. Collectively, these findings hinted that comparative analysis of hepatic metabolites across species represents a valuable strategy for elucidating the mechanisms underlying interspecies variations in gluconeogenic efficiency.

The rate of gluconeogenesis is primarily regulated by key enzymes, including fructose 1,6-bisphosphatase (FBP1/2), phosphoenolpyruvate carboxykinase (PCK1/2), and glucose 6-phosphatase (G6PC) [5]. The expression of these rate-limiting enzymes mainly relies on some transcription factors, such as cAMP-response element binding protein (CREB), hepatic nuclear receptor 4α (HNF4α), forkhead box O1 (FOXO1), and peroxisome proliferator-activated receptor-y coactivator-1α (PGC-lα) [5–7]. In addition, the transcriptional activity of CREB is regulated by multiple mechanisms. One such mechanism involves G protein-coupled receptors (GPCRs) being activated by signaling molecules, which leads to the conversion of more cytoplasmic ATP into cAMP. This subsequently activates downstream protein kinase A (PKA). Activated PKA phosphorylates CREB and dephosphorylates CRTC2 [8]. The phosphorylated CREB then combines with dephosphorylated CRTC2 to form a complex, which jointly promotes the expression of downstream rate-limiting enzymes [9].

During gluconeogenesis, the activity of transcription factors and rate-limiting enzymes is modulated by various regulators. Beyond established hormonal regulators such as insulin and glucagon [10, 11], specific metabolites, including amino acids, fatty acids, and bile acids, also influence hepatic gluconeogenesis. For instance, histidine supplementation stimulates gluconeogenesis in the dairy cow liver by upregulating key gluconeogenic genes [12]. Additionally, alanine supplementation activates gluconeogenesis [13]. Propionic acid enhances the expression of key gluconeogenic rate-limiting enzymes in dairy cow liver cells via the mTOR signaling pathway, consequently increasing lactose production [6, 14]. Among bile acids, certain types modulate gluconeogenesis by either promoting or inhibiting the expression of rate-limiting enzymes [15], whereas the roles of others remain to be elucidated.

Bile acids are secreted by the liver and stored in the gallbladder. They are primarily categorized into two types: primary bile acids and secondary bile acids. Primary bile acids are synthesized in the liver and exhibit species-specific variations in their synthesis pathways [16]. For instance, in dairy cows, cholic acid (CA) and chenodeoxycholic acid (CDCA) constitute the predominant primary bile acids, accounting for up to 80% of the primary or primary-conjugated bile acids present in the liver [17]. Within the liver, primary bile acids undergo conjugation with glycine or taurine, forming conjugated primary bile acids such as glycocholic acid (GCA) and taurocholic acid (TCA) [18]. These bile acids enter the intestine, where they are metabolized by gut microorganisms to form secondary bile acids [19]. While a minor fraction of secondary bile acids is excreted, the majority undergoes enterohepatic circulation, being reabsorbed and transported back to the liver. Here, they can be further processed, including re-conjugation, to form bile acids such as taurodeoxycholic acid (TDCA) [18].

Bile acids mediate their physiological effects through interactions with multiple receptors. Key receptors include nuclear receptors such as the farnesoid X receptor (FXR) and the membrane-bound Takeda G protein-coupled receptor 5 (TGR5) [20]. TGR5 is expressed in multiple tissues, including the liver, kidneys, heart, skeletal muscle, and placenta [21]. This receptor plays a critical role in regulating blood glucose homeostasis, ameliorating obesity, modulating intestinal microbiota composition, and suppressing appetite via hypothalamic signaling pathways [22–24]. Current studies have demonstrated that bile acids can increase the expression of PCK by inhibiting FXR, thereby promoting gluconeogenesis in hepatocytes [25]. However, the specific bile acids that regulate gluconeogenesis via TGR5 signaling remain to be elucidated.

Therefore, by comparing two species with differing efficiencies of liver gluconeogenesis, we sought to identify potential metabolites that modulate hepatic gluconeogenesis and elucidate their mechanisms of action by comparing species exhibiting divergent gluconeogenic efficiencies. This would enhance our understanding of the nutritional regulation of liver gluconeogenesis in dairy cows. Furthermore, we validated the regulatory potential of these newly identified metabolites using murine models, with the future objective of validating and applying these insights in cows to develop nutritional strategies that improve liver gluconeogenesis in dairy cows and increase milk production efficiency.

## Materials and methods

### Cattle and pig studies

#### Liver tissue sample collection

All animal procedures were approved by the Animal Care Committee of Northwest A&F University. In this study, animals were selected from a commercial dairy farm and a commercial pig farm in Shaanxi Province, including six Holstein dairy cows and six Duroc × (Landrace × Yorkshire) (DLY) crossbred pigs. The Holstein cows were in the dry period of their third lactation, and the multiparous hybrid pigs were in the non-pregnant phase of their second lactation. Individuals within the same species were matched for body condition, age, and parity. All experimental animals were confirmed absence of pathologies. Liver specimens were collected immediately post-slaughter for metabolomic analysis. Samples were snap-frozen in liquid nitrogen within minutes of collection and stored at −80 °C until analysis.

#### Untargeted and targeted metabolomics

A 20 mg Liver tissue sample was mixed with two magnetic beads, followed by the addition of 10 μL of working solution (containing 10 μg/mL internal standard) and 200 μL of a 20% (v/v) methanol–acetonitrile mixture. The mixture was vortexed at 538 × *g* for 10 min, incubated at −20 °C for 10 min, and then centrifuged at 4 °C and 12,396 × *g* for 10 min. The supernatant was collected and re-dissolved in 100 μL of 50% methanol-water solution for subsequent analysis. The LC-MS/MS analysis was performed using an ultra-performance liquid chromatography (UPLC) system (ExionLC™ AD) coupled with a tandem mass spectrometry (MS/MS) system (QTRAP^®^ 6500+). The liquid chromatography conditions were as follows: the chromatographic column was a Waters ACQUITY UPLC HSS T3 C18 column (1.8 μm, 100 mm × 2.1 mm i.d.). Mobile phase A consisted of ultrapure water containing 0.01% acetic acid and 5 mmol/L ammonium acetate, while mobile phase B was acetonitrile containing 0.01% acetic acid. The flow rate was set to 0.35 mL/min, the column temperature was maintained at 40 °C, and the injection volume was 3 μL. Gradient elution was performed according to the following program: initial A/B ratio of 95:5 (v/v), adjusted to 60:40 (v/v) after 0.5 min, 50:50 (v/v) at 4.5 min, 25:75 (v/v) at 7.5 min, 5:95 (v/v) at 10 min, and restored to 95:5 (v/v) at 12.0 min. Mass spectrometry conditions were as follows: electrospray ionization (ESI) source temperature was set to 550 °C, ion spray voltage was −4500 V, curtain gas (CUR) pressure was 35 psi. In the triple quadrupole mass spectrometer, each target ion pair was detected based on its optimized declustering potential (DP) and collision energy (CE).

### Mouse studies

#### Animals

All experimental mice in this study were approved by the Institution Animal Care and Use Committee of the Northwest A&F University (approval No. NWAFU‐2020‐1131). Male C57BL/6J mice (8 weeks old, Beijing Huafukang Biotechnology Co., Ltd., Beijing, China) were housed in a specific pathogen-free (SPF) environment with a 12:12 light-dark cycle, at an ambient temperature of 24 °C (± 1 °C), and relative humidity of 50% to 60%. Mice were provided ad libitum access to feed and sterile water. The home cages were equipped with sufficient bedding, which was replaced every 3 d to maintain a clean and comfortable environment.

#### BAs treatment

Fourteen mice were randomly divided into two groups. One group was provided with drinking water, while the other group received drinking water supplemented with bile acids (BAs). The specific composition of BAs was a mixture of 0.5% w/v sodium taurodeoxycholate (207737-97-1; Psaiton, Beijing, China), sodium taurocholate (A601143; Sangon, Shanghai, China), and sodium glycocholate (MB3541; MelunBio, Dalian, China) dissolved in ddH_2_O at a 1:1:1 ratio. Mice were fed daily throughout the entire process, which lasted for 3 weeks.

#### TGR5 inhibitor treatment

The TGR5 inhibitor SBI-115 (GC34775, Gipbio, Montclair, CA, USA) was dissolved in corn oil containing 5% dimethylsulfoxide (DMSO). This experiment employed a 2 × 2 factorial design, with BAs and SBI-115 serving as the dependent variables, thereby comprising a total of four groups. Each group consisted of 7 mice. In the SBI-115 treatment group, mice were intraperitoneally injected with 200 µL of corn oil containing 100 mg/kg of SBI-115 every 2 d.

#### Measurement of fasting blood levels of glucose

Fasting glucose was measured using a blood glucose meter (Exactive EQ lmpulse, MicroTech Medical, Hangzhou, China) after 12 h of fasting.

#### Glucose tolerance test and pyruvate tolerance test

The glucose tolerance test (GTT) or pyruvate tolerance test (PTT) was performed after 12 h of fasting. Blood glucose levels were measured at 0, 15, 30, 60, 90, and 120 min following intraperitoneal injection of 1.5 g/kg glucose for GTT or 1.5 g/kg pyruvate for PTT.

### Cell studies

#### Cell culture

The bovine hepatocyte (BH) was provided by Professor Jianguo Wang's laboratory at the College of Veterinary Medicine, Northwest A&F University. BH was confirmed to express both bovine serum albumin (BSA) and cytokeratin 18 (CK18), which indicated that this cell line exhibited both the functional characteristics of hepatocytes and the phenotypic features of immortalized epithelial cell lines. The HEK293T cell line was purchased from Procell (Wuhan, China). Both cell lines were maintained in Dulbecco's Modified Eagle Medium (DMEM, Gibco, Grand Island, NY, USA) supplemented with 5% fetal bovine serum (FBS, Gibco, Grand Island, NY, USA). Cells were cultured in a humidified incubator at 37 °C with 5% CO₂.

#### BAs treatment and inhibitor treatment

The BH were treated with TDCA (T0875), TCA (T4009), GCA (700265P), or mixed bile acids. These were purchased from Sigma-Aldrich (St. Louis, MO, USA) Additionally, 10 μmol/L H-89 (S1582, Selleck Chemicals, Houston, TX, USA), 17 μmol/L Guggulsterone (GUG, S6812, Selleck Chemicals, Houston, TX, USA), or 10 μmol/L SBI-115 (882366-16-7, Medchemexpress, NJ, USA) was added to the culture medium to inhibit homologous protein activity. After 12 h of treatment, the cell samples were lysed using loading buffer and collected for subsequent Western blot analysis.

#### Plasmid and siRNA transfection

The cells were transfected when the cell density reached 50%–60%. For plasmid transfection, the negative control pcDNA3.1(+), Flag-CREB, and HA-CRTC2 plasmids were mixed with polyethylenimine (PEI; 919012, Sigma-Aldrich, St. Louis, MO, USA) and incubated in serum-free medium for 30 min. In another set of plasmid transfections, the pGL3-FBP1/PCK1/G6PC and pRL-TK plasmids were mixed with Lipofectamine 2000 (18324012, Invitrogen, Carlsbad, CA, USA) and incubated in serum-free medium for 30 min. Cells transfected with pGL-Basic plasmid served as the negative control. For siRNA experiments, the negative control siRNA or TGR5 siRNA (GenePharma, Suzhou, China) was mixed with Lipofectamine 2000 and incubated in serum-free medium for 30 min. After a 6-h incubation, the medium was replaced with complete growth medium.

The following siRNA were used:

- siTGR5: 5ʹ‐GCGUGGACCUUGACUUGAA‐3ʹ.

#### Western blot

Mouse liver tissue samples were homogenized in RIPA lysis buffer for protein extraction. Protein concentration was quantified using a BCA assay kit (QPBCA, Sigma-Aldrich, USA). Briefly, 25 μL of QuantiPro Buffer QA (M3810), 25 μL of QuantiPro BCA QB (M3685), and 1 μL of 4% Copper(II) sulfate pentahydrate solution (C2284) were mixed in a 96-well microplate. Subsequently, 0.5 μL of each protein sample was added to the mixture and incubated at 37 °C for 2 h. After incubation, the plate was equilibrated to room temperature, and absorbance was measured at 562 nm using a microplate reader. The cells were washed with PBS, and then suspended in loading buffer added β-mercaptoethanol for 5 min. The proteins were separated by SDS-PAGE and transferred onto nitrocellulose membranes (0.45 μmol/L, GE). The membranes were blocked with 5% BSA powder in phosphate-buffered saline and then incubated at 4 °C overnight with the primary antibody. The primary antibodies FBP1 (12842-1-AP), FBP2 (25192-1-AP), PCK1 (16754-1-AP), PCK2 (14892-1-AP), G6PC (66860-1-Ig), p-CREB (28792-1-AP), PGC-1α (66369-1-Ig), CREB (67927-1-Ig), β-actin (66009-1-Ig), HA (51064-2-AP) were purchased from Proteintech (Wuhan, China) and HNF-4α (db15987), Flag (db7002) were purchased from Diagbio Biosciences (Hangzhou, China). After three washes with phosphate-buffered saline with 0.1% Tween-20 (PBST; Sangon, Shanghai, China), the membranes were incubated with secondary antibodies: rabbit anti-mouse (AP160P) and goat anti-rabbit (AP132) purchased from Sigma-Aldrich (St. Louis, MO, USA) for 2 h at room temperature, followed by 3 washes with PBST. Protein detection was performed using the Bio-Rad Imaging System (Bio-Rad, Hercules, TX, USA). All the antibodies were diluted according to the dilution ratio specified in the instructions.

#### RT-qPCR

After the cells were washed with PBS, TRIzol reagent (DP424, TIANGEN, Beijing, China) was used to extract total RNA. Chloroform and isopropanol were sequentially added for phase separation and RNA precipitation, followed by washing with anhydrous ethanol and drying. The RNA was dissolved in DEPC-treated water, and its purity (OD_260/280_) and concentration were measured using a spectrophotometer (Thermo Fisher Scientific, Waltham, MA, USA). First-strand cDNA synthesis was performed using the HiScript II First Strand cDNA Synthesis Kit (R212-02, Vazyme, Nanjing, China). For RT-qPCR analysis, the reaction mixture consisted of 5 μL ChamQ Universal SYBR qPCR Master Mix (Q711-02, Vazyme, Nanjing, China), 0.5 μL of each forward and reverse primer, and 4 μL of the cDNA template. The thermal cycling conditions were as follows: initial denaturation at 95 °C for 30 s, followed by 40 cycles of denaturation at 95 °C for 10 s, annealing at 60 °C for 30 s. Gene expression levels were normalized to β-actin and quantified using the 2^-ΔΔCT^ method. The RT-qPCR primers were designed and their sequences were available in the NCBI database (https://www.ncbi.nlm.nih.gov), as shown in Table S1 (Additional file 1).

#### Measurement of glucose production

When the cell density reached 50%–60%, the BH medium was replaced with a glucose-free medium (11966025, Gibco, Grand Island, USA) supplemented with 5 μmol/L pyruvate. After 6 h of incubation, the culture medium was centrifuged at 12,369 × *g* for 5 min, and the supernatant was collected. The glucose concentration in the culture medium was measured using a Glucose Assay Reagent (S0201S, Beyotime, Shanghai, China). The procedure involved heating a solution containing 15 μL of supernatant and 185 μL of glucose determination reagent at 95 °C for 8 min, followed by cooling to 4 °C. Subsequently, 190 μL of the reaction solution was transferred to a 96-well plate, and the optical density was measured at 630 nm using an enzyme marker (TECAN, Spark, Switzerland). The glucose concentration was calculated based on the standard curve.

#### Measurement of cAMP concentration

A 96-well microplate was prepared with designated wells for standards, blanks, and samples. The following additions were performed: standard wells: 50 μL of standard dilutions; blank wells: 50 μL of assay diluent; sample wells: 50 μL of test samples. Subsequently, 50 μL of prepared working solution was dispensed into all wells. The plate was sealed and incubated at 37 °C for 45 min. After incubation, liquid was decanted and residual moisture removed by blotting on absorbent filter paper. Each well was washed with 350 μL washing buffer (1 min immersion), followed by decanting and blotting. The wash cycle was repeated three times. Immediately post-wash, 100 μL of enzyme-conjugate working solution was added to each well. The sealed plate was incubated at 37 °C for 30 min. Following conjugate removal, five additional wash cycles were performed as described. Substrate solution (90 μL/well) was added, and the plate was incubated under light-protected conditions at 37 °C for 15 min. Reactions were terminated by adding 50 μL stop solution per well. Optical density (OD) was measured immediately at 450 nm using a microplate reader.

#### Co-Immunoprecipitation

After the cells were washed with PBS, they were lysed in a freeze lysis buffer containing 2% phosphatase inhibitors (Na_3_VO_4_ and NaF) and the 1% protease inhibitor phenylmethylsulfonyl fluoride (PMSF) for 30 min, followed by centrifugation at 14,972 × *g* for 15 min. The cell lysate was incubated with anti-Flag M2 beads (A2220, Sigma-Aldrich, St. Louis, USA) for 6–8 h at 4 °C. Non-specifically bound proteins were removed by washing with lysis buffer, and the supernatant was discarded. The washed precipitate was retained, and 2 × loading buffer was added. Protein binding was subsequently detected by Western blot analysis.

#### Dual luciferase assay

Dual luciferase assay was performed as previously described [26]. The Dual-Luciferase Reporter Assay Kit (RG027, Beyotime, Shanghai, China) was used to detect the activation of promoter. Firefly luciferase-derived relative light units (RLU) were normalized to Renilla luciferase RLU values, which served as an internal control. Comparative analysis of reporter gene activation across experimental groups was performed based on the calculated normalized RLU ratios.

### Statistical analysis

The raw LC-MS data were processed and converted into mzML format using ProteoWizard software. Subsequently, peak extraction, alignment, and retention time correction were performed with the XCMS program. Our analytical methodology was predominantly informed by prior research findings [27]. The XCMS was freely available under an open-source license at https://metlin.scripps.edu/download. The peak areas were normalized via the support vector regression (SVR) algorithm, and peaked with missing values exceeding 50% across all samples were excluded. Metabolite identification was achieved by integrating internal laboratory databases, public resource databases, artificial intelligence prediction databases, and the metDNA method. In the statistical analysis stage, R software was employed for in-depth processing: the data were standardized using unit variance scaling (UV Scaling), and principal component analysis (PCA) was conducted using the prcomp function. Differential metabolites were identified by combining the variable importance in projection (VIP > 1) from the orthogonal partial least squares discriminant analysis (OPLS-DA) model with Student's *t*-test (*P* < 0.05). The OPLS-DA results were visualized through score plots and permutation test plots. All analyses were conducted using the MetaboAnalystR package, and the data were log2-transformed and centered prior to OPLS-DA. Hierarchical clustering analysis of samples and metabolites was performed using the ComplexHeatmap package, and the results were presented as heat maps and dendrograms. The Pearson correlation coefficient between samples was calculated using the built-in cor function in R and visualized. Finally, the KEGG compound database was queried for metabolite identification, and these metabolites were mapped to the KEGG pathway database for enrichment analysis. Significant metabolic pathways were determined based on the hypergeometric distribution test.

All statistical analyses were performed using GraphPad Prism 8.0. Data are expressed as mean ± SEM. Statistical comparisons employed unpaired Student's *t*-test and one-way analysis of variance (ANOVA). Statistical significance was defined as *P* < 0.05.

## Results

### Bile acids were identified as key gluconeogenesis regulators

**Fig. 1:**
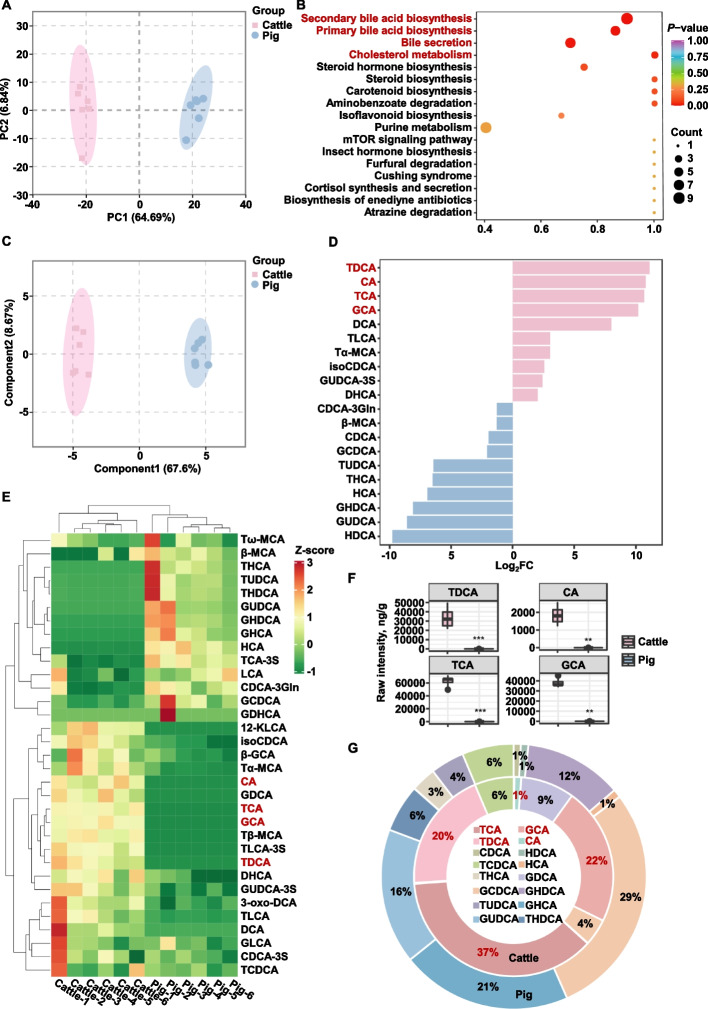
Bile acids were identified as key gluconeogenesis regulators. **A** Principal component analysis (PCA) of non-targeted metabolomics data from Holstein dairy cows (*n* = 6) and DLY pigs (*n* = 6). **B** KEGG pathway enrichment analysis of differential metabolites identified from non-targeted metabolomics. **C** Orthogonal partial least squares discriminant analysis (OPLS-DA) based on targeted bile acid metabolomics. **D** Top 10 and bottom 10 bile acids ranked by fold change between Holstein cows and DLY pigs. **E** Clustering heatmap of bile acid contents across all liver samples. **F** Absolute concentrations of the top four bile acids with the most significant fold changes (TDCA, CA, TCA, and GCA). **G** Proportions of individual bile acids relative to total bile acids in Holstein cow liver samples. All data were represented as mean ± SEM. ^*^P < 0.05, ^**^P < 0.01 and ^***^P < 0.001 compared to the CON group

To elucidate mechanisms governing the divergent hepatic gluconeogenesis capacities between ruminants and monogastric species, untargeted metabolomics was performed on liver specimens from Holstein dairy cows and DLY crossbred pigs, identifying candidate metabolites modulating this metabolic pathway. Non-targeted metabolomic profiling was performed on liver samples from Holstein cows and DLY crossbred pigs, revealing a clear separation between the principal components (PCA) of the two groups (Fig. 1A), indicating distinct liver metabolomic profiles in dairy cows and pigs. Differential metabolites were quantitatively analyzed using volcano plots, and fold changes in liver metabolite levels were visualized (Fig. S1A and B). Subsequently, differential metabolites identified in the two groups were annotated and mapped using the KEGG database, which revealed that the major metabolic pathways involved were secondary bile acid biosynthesis and primary bile acid biosynthesis (Fig. 1B). These results suggested that the bile acid pathway may play a critical role in regulating gluconeogenesis function. Therefore, targeted bile acid metabolomics analysis was conducted on liver samples from both groups. Orthogonal partial least squares discriminant analysis (OPLS-DA) demonstrated significant differences in bile acid metabolites between dairy cow and pig liver tissues. Quantitative analysis of bile acid concentrations was performed, and fold differences were visualized using bar charts (Fig. 1D and Fig. S1C). The results showed that taurodeoxycholic acid (TDCA), cholic acid (CA), taurocholic acid (TCA), and glycocholic acid (GCA) were the top four bile acids with higher content in dairy cow liver compared to pig liver. Bile acid profiles exhibited consistent inter-sample homogeneity, with distinct clustering patterns observed in multivariate analysis (Fig. 1E). Specifically, TDCA, TCA, and GCA accounted for 37%, 22%, and 20% of total bile acids in dairy cow liver, respectively, while CA accounted for only 1% (Fig. 1G). Bile acid profiles demonstrated consistent homogeneity across all samples (Fig. S1D). Specifically, the absolute concentrations of TDCA, TCA, and GCA were significantly elevated in dairy cow liver tissues compared to porcine specimens (Fig. 1F). Based on these findings, TDCA, TCA, and GCA appeared to differ significantly between dairy cow and pig liver tissues.

### TDCA, TCA, and GCA promoted gluconeogenesis in bovine liver cells

**Fig. 2:**
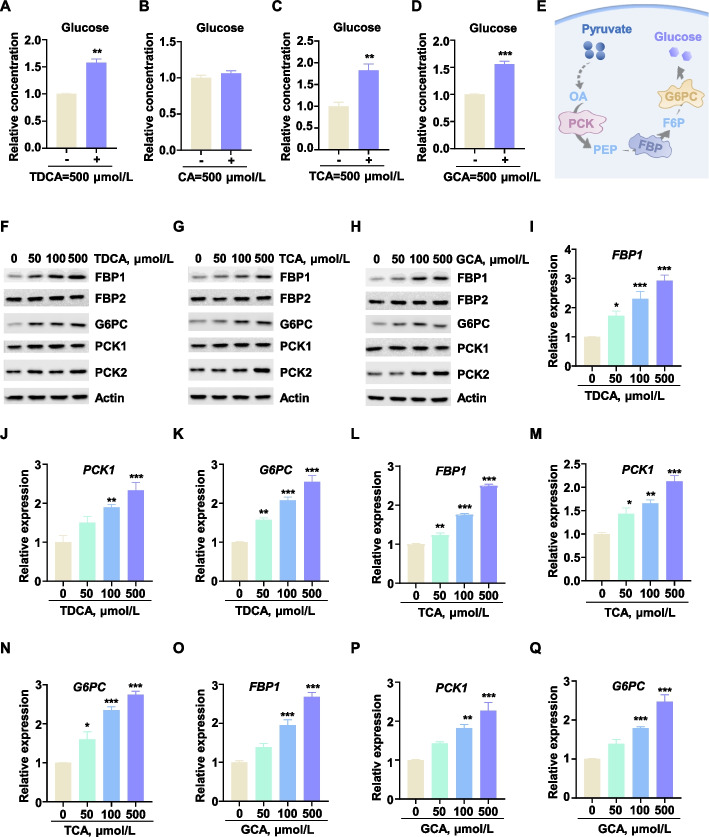
TDCA, TCA, and GCA promoted gluconeogenesis in bovine liver cells. **A–D** Add TDCA, CA, TCA, or GCA separately to detect their effects on glucose production in BH (*n* = 3). **E** Metabolic process model of pyruvate-to-glucose conversion. **F–H** Protein expression levels of gluconeogenesis rate-limiting enzymes after BH stimulation by TDCA, TCA, or GCA. **I–Q** The mRNA expression levels of gluconeogenesis rate-limiting enzymes after BH stimulation by TDCA, TCA, or GCA (*n* = 3). All assays were carried out in at least three independent biological replicates. All data were represented as mean ± SEM. ^*^P < 0.05, ^**^P < 0.01 and ^***^P < 0.001 compared to the CON group

To assess the regulatory effects of the identified bile acids on hepatic gluconeogenesis in ruminants, we added TDCA, CA, TCA, and GCA to the bovine hepatocyte (BH) individually and measured glucose production in BH. TDCA, TCA, and GCA significantly stimulated glucose output in BH, whereas CA exhibited no significant effect (Fig. 2A–D). Given that hepatic gluconeogenesis replenishes glucose reserves through pyruvate, catalyzed by rate-limiting enzymes including PCK, FBP, and G6PC (Fig. 2E), we interrogated bile acids effects on this pathway by assessing expression of these regulatory enzymes in BH. We observed that exposure of BH to increasing concentration gradients of TDCA, TCA, and GCA led to a dose-dependent upregulation of the protein expression levels of FBP1, PCK1, and G6PC, while the protein expression levels of FBP2 and PCK2 remained unchanged (Fig. 2F–H), and the mRNA expression patterns were consistent with these findings (Fig. 2I–Q and Fig. S2A–F). In contrast, CA exhibited no significant effect on the gluconeogenesis capacity of BH (Fig. S2G–L), suggesting that it was not the key metabolite in the gluconeogenesis process. Therefore, TDCA, TCA, and GCA effectively promoted gluconeogenesis in BH by upregulating the expression of the rate-limiting enzyme genes *FBP1*, *PCK1*, and *G6PC*.

**Fig. 3:**
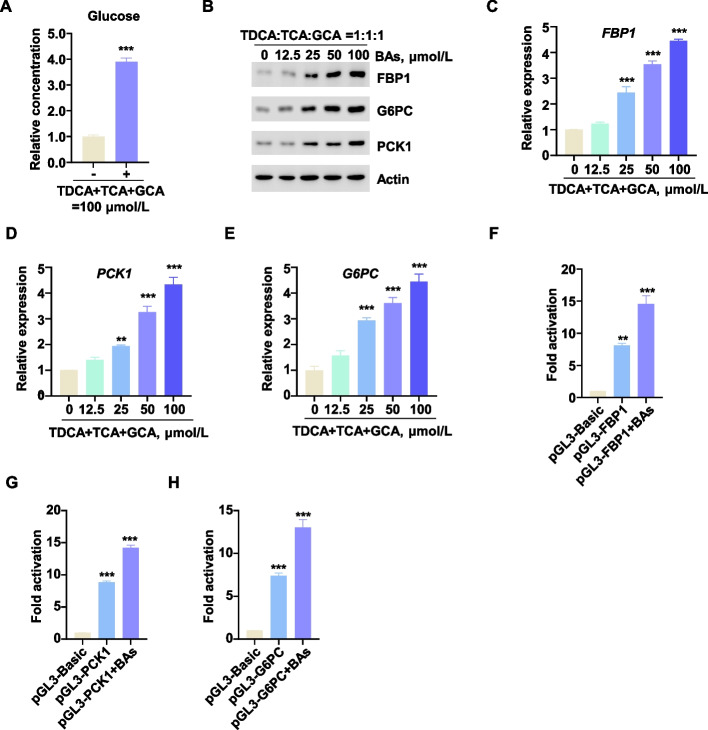
Effects of mixed bile acids (TDCA, TCA, and GCA) on hepatic gluconeogenesis. **A** Mix and add TDCA, TCA, and GCA (BAs), followed by detecting glucose production in BH (*n* = 3). **B** The protein expression levels of FBP1, PCK1 and G6PC induced by BAs. **C–E** Effects of BAs on mRNA expression levels of* FBP1*, *PCK1* and *G6PC* (*n* = 3). **F–H** Dual-luciferase reporter gene assays to evaluate the influence of BAs on the transcriptional activity of *FBP1*, *PCK1* and *G6PC* (*n* = 3). All assays were carried out in at least three independent biological replicates. All data were represented as mean ± SEM. ^*^P < 0.05, ^**^P < 0.01 and ^***^P < 0.001 compared to the CON group

To model physiologically relevant synergistic interactions within the bovine hepatic milieu, combinatorial treatment with the three efficacious bile acids (TDCA, TCA, and GCA) was administered to assess their collective impact on gluconeogenesis. Relative to single-agent treatments, the bile acid mixture (hereinafter referred to as BAs) significantly enhanced gluconeogenesis in BH at reduced concentrations. This synergistic effect was evidenced by elevated glucose output and upregulated expression of key enzymatic genes *FBP1*, *PCK1*, and *G6PC*. (Fig. 3A–E). Concurrently, a dual-luciferase reporter system targeting bovine *FBP1*, *PCK1*, and *G6PC* promoters was employed. BAs significantly transactivated these promoters (Fig. 3F–H). These results suggested that TDCA, TCA, and GCA collectively enhanced gluconeogenesis in dairy cow livers.

### Bile acids enhanced liver gluconeogenesis in vivo

**Fig. 4:**
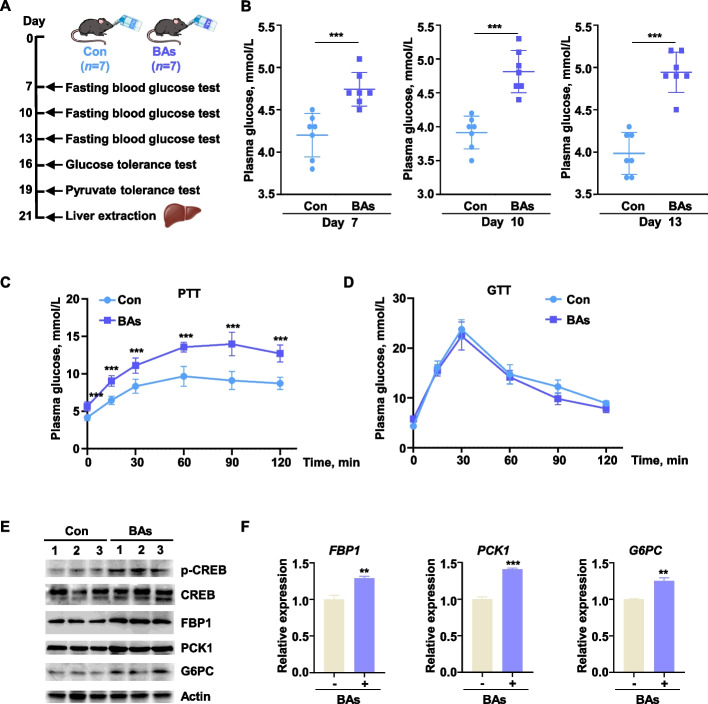
Bile acids enhanced liver gluconeogenesis in vivo. **A** Flowchart of the mouse feeding experiment with BAs. **B** Fasting blood glucose levels in mice (*n* = 7) on d 7, 10, and 13 after BAs feeding. **C** Glucose tolerance in mice (*n* = 7) on d 16 after BAs feeding. **D** Pyruvate tolerance in mice (*n* = 7) on d 19 after BAs feeding. **E** and **F** Protein expression levels and mRNA expression levels of in Liver tissue samples collected from mice on the 21st day after BAs feeding. All data were represented as mean ± SEM. ^*^P < 0.05, ^**^P < 0.01 and ^***^P < 0.001 compared to the CON group

A significant correlation was observed between the BA mixture and hepatic gluconeogenesis efficiency. Nevertheless, it remained unclear whether these mixed bile acids could regulate systemic gluconeogenesis capacity. To investigate this, we performed an in vivo study in mice, administering BAs for three weeks (Fig. 4A). On d 7, 10, and 13 of administration, fasting blood glucose levels were assessed. Significantly elevated fasting blood glucose was observed in BAs-treated mice compared to controls (Fig. 4B). The pyruvate tolerance test (PTT) serves as a critical indicator of hepatic gluconeogenic capacity, specifically reflecting the liver's ability to convert pyruvate into glucose. Our results demonstrated that TDCA, TCA, and GCA significantly enhanced pyruvate tolerance in fasted mice. This enhancement was directly associated with an increased efficiency of hepatic pyruvate-to-glucose conversion, indicative of stimulated gluconeogenesis (Fig. 4C). Conversely, glucose tolerance tests (GTT) revealed that these bile acids exerted no discernible effect on glucose tolerance in mice, suggesting a specific regulatory role in gluconeogenesis (Fig. 4D). Western blot and qPCR analyses confirmed significant upregulation of both protein and mRNA expression levels for the key gluconeogenic enzymes PCK1, FBP1, and G6PC in the livers of BAs-treated mice (Fig. 4E and F). Collectively, these findings indicated that BAs enhanced hepatic gluconeogenesis in mice.

### BAs promoted CREB phosphorylation and CRTC2 binding via the cAMP/PKA pathway

**Fig. 5:**
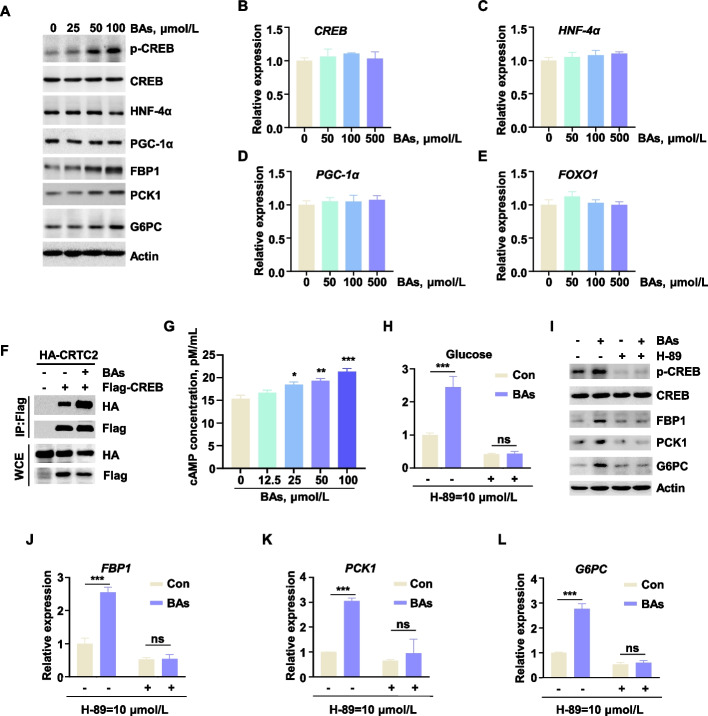
BAs promoted CREB phosphorylation and CRTC2 binding via the cAMP/PKA pathway. **A** Protein expression levels of transcription factors regulating gluconeogenesis rate-limiting enzymes in BH after adding BAs to the medium. **B–E** mRNA expression levels of transcription factors *CREB*, *HNF-4α*, *PGC-1α* and *FOXO1* in BH after adding BAs to the medium (*n* = 3). **F** Binding situation of exogenous CREB and CRTC2 under BAs stimulation (100 μmol/L). **G** cAMP concentration in BH after adding BAs to the medium at a concentration gradient (*n* = 3). **H** Glucose production in BH stimulated by BAs (100 μmol/L) after treatment with the PKA inhibitor H-89 (*n* = 3). **I** Protein expression levels in BH under BAs stimulation (100 μmol/L) after using H-89. **J–L** mRNA expression levels of *FBP1*, *PCK1*, and *G6PC* in BH after using H-89 under BAs (100 μmol/L) stimulation (*n* = 3). All assays were carried out in at least three independent biological replicates. All data were represented as mean ± SEM. ^*^P < 0.05, ^**^P < 0.01 and ^***^P < 0.001 compared to the CON group

To investigate the mechanism by which BAs regulate hepatic gluconeogenesis in dairy cows, we examined key transcription factors associated with the expression of genes encoding rate-limiting gluconeogenic enzymes. The results demonstrated that BA administration promoted CREB phosphorylation, while exerting no significant effect on the expression levels of other transcription factors, including HNF4α, PGC-1α, and FOXO1 (Fig. 5A–E). Furthermore, assessment of p-CREB levels in liver tissues from mice administered BAs yielded consistent findings (Fig. 4E).

Given the stimulatory effects of BAs on gluconeogenesis and their impact on the transcription factor p-CREB, we further investigated the underlying mechanism. Flag-CREB and HA-CRTC2 were transfected into HEK293T cells, and the interaction between CREB and CRTC2 following BAs treatment was evaluated using co-immunoprecipitation. As anticipated, BAs treatment enhanced the CREB-CRTC2 interaction (Fig. 5F), indicative of increased transcriptional complex formation. Subsequently, dual-luciferase reporter assays were conducted to assess the interaction between transcription factors and the promoter regions of genes encoding rate-limiting gluconeogenic enzymes. As shown in Fig. 3F–H, BAs significantly enhanced the transcriptional activity of the *FBP1*, *PCK1* and *G6PC* promoters, thereby promoting their expression. These results demonstrated that BAs promoted CREB phosphorylation and facilitate its interaction with CRTC2, leading to the transcriptional activation of key gluconeogenic enzyme genes and enhanced hepatic gluconeogenesis in dairy cows.

Given that CREB phosphorylation is mediated by the cAMP/PKA signaling pathway, we hypothesized that BA-induced CREB phosphorylation might involve cAMP/PKA activation. To test this hypothesis, BH were treated with BAs in the presence of a PKA inhibitor, followed by assessment of CREB phosphorylation and expression of rate-limiting gluconeogenic enzymes. The cAMP quantification revealed that BAs significantly elevated intracellular cAMP levels. Specifically, BAs treatment increased cAMP concentrations in BH by 39% relative to untreated controls (Fig. 5G). Additionally, PKA inhibition suppressed CREB phosphorylation and reduced FBP1, PCK1, and G6PC expression levels, thereby abolishing the stimulatory effects of BAs on gluconeogenesis (Fig. 5H–L). These findings indicated that BAs activate CREB phosphorylation via the cAMP/PKA signaling pathway, consequently regulating hepatic gluconeogenesis in dairy cows.

### TGR5 was the key mediator of bile acid induced gluconeogenic activation

**Fig. 6:**
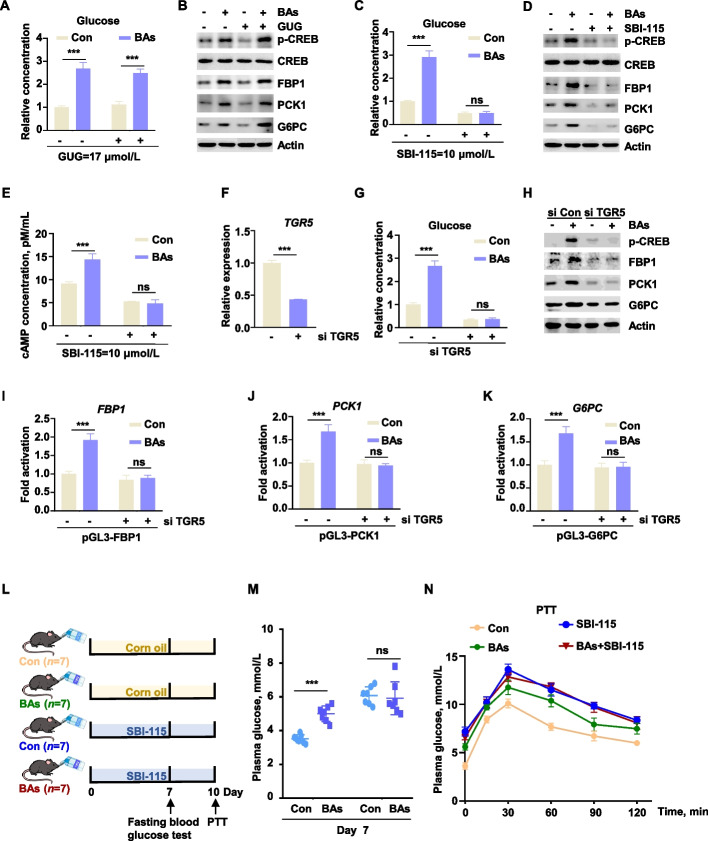
The TGR5 was the key mediator of bile acid-induced gluconeogenic activation. **A** The glucose production after treatment with the FXR inhibitor guggulsterone (GUG) and BAs (100 μmol/L) (*n* = 3). **B** The protein expression levels in BH were detected under BAs (100 μmol/L) and GUG stimulation. **C** The glucose production after treatment with the TGR5 inhibitor SBI-115 and BAs (100 μmol/L) (*n* = 3). **D** The protein expression levels in BH were detected under BAs (100 μmol/L) and SBI-115 stimulation. **E** The cAMP concentration in BH were detected with BAs (100 μmol/L) and SBI-115 treatment (*n* = 3). **F** Knockdown efficiency of *TGR5* by siRNA (*n* = 3). **G** After knocking down *TGR5* (siTGR5) with siRNA, the glucose production was detected under BAs (100 μmol/L) stimulation (*n* = 3). **H** The protein expression levels in BH with BAs (100 μmol/L) and siTGR5 treatment. **I–K** The transcriptional activities of *FBP1*, *PCK1* and *G6PC* with BAs (100 μmol/L) and siTGR5 treatment (*n* = 3). **L** Experimental flowchart for intraperitoneal injection of SBI-115 in BAs-fed mice. **M** Fasting blood glucose (*n* = 7) levels in BAs-fed mice on d 7. **N** Pyruvate tolerance (*n* = 5) in BAs-fed mice on d 10. All assays were carried out in at least three independent biological replicates in **A–K**. All data were represented as mean ± SEM. ^*^P < 0.05, ^**^P < 0.01 and ^***^P < 0.001 compared to the CON group

As endogenous ligands, bile acids bind to multiple receptors, among which FXR and TGR5 represent key mediators. To delineate receptor-specific contributions to BAs induced hepatic gluconeogenesis, we inhibited the receptors of bile acids in BH. Pharmacological inhibition of TGR5 with SBI-115 or FXR with guggulsterone (GUG) revealed that TGR5 blockade significantly attenuated glucose production stimulated by BAs, whereas FXR inhibition exerted no significant effect (Fig. 6A and C). Inhibiting FXR activity with the inhibitor GUG did not affect the action of BAs (Fig. 6B and Fig. S3A–C). TGR5 inhibition abolished BA-induced upregulation of rate-limiting enzyme expression and CREB phosphorylation at both protein and mRNA levels (Fig. 6D and Fig. S3D–F). Moreover, TGR5 blockade reduced intracellular cAMP concentrations in BH (Fig. 6D and Fig. S3D–F). Furthermore, the cAMP concentration in BH decreased after TGR5 inhibition (Fig. 6E). These findings demonstrated that TGR5 mediated the regulation of gluconeogenesis by sensing BAs and influencing downstream cAMP signaling.

To validate these findings, we performed *TGR5* knockdown using siRNA (Fig. 6F). Intuitively, a reduction in glucose production was observed in *TGR5* knockdown cells (Fig. 6G). Consistent with pharmacological inhibition, genetic ablation of *TGR5* abolished the stimulatory effects of BAs on gluconeogenesis. Specifically, TGR5 silencing attenuated CREB phosphorylation and decreased protein expression of the rate-limiting enzymes FBP1, PCK1, and G6PC (Fig. 6H). Additionally, qPCR and dual-luciferase reporter assays confirmed that the transcription of these genes were inhibited (Fig. 6I–K and Fig. S3G–I). Moreover, TGR5 inhibition in mice did not alter fasting blood glucose levels or PTT outcomes under BA treatment conditions (Fig. 6L–N), though the inhibitor SBI-115 induced systemic hyperglycemia potentially through extrahepatic mechanisms. Collectively, these results demonstrated that BAs enhanced gluconeogenic capacity through the TGR5/cAMP/PKA/CREB axis, which upregulated transcription and expression of key gluconeogenic enzymes.

## Discussion

**Fig. 7:**
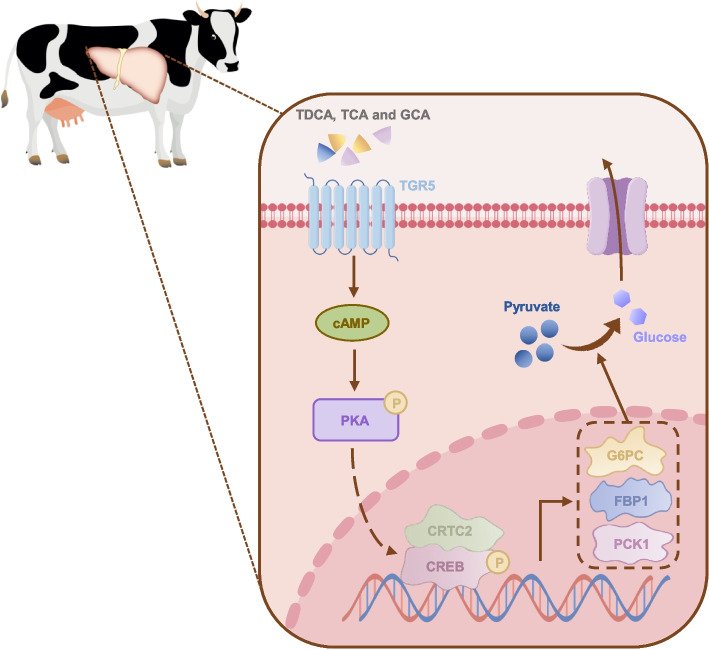
Promotion of hepatic gluconeogenesis in dairy cows by bile acids TDCA, TCA and GCA via TGR5. TDCA, TCA and GCA activated TGR5, elevated intracellular cAMP levels, and ultimately enhanced gluconeogenesis via the CREB

Glucose serves as the primary energy source for maintenance and lactose synthesis in dairy cows, and its synthesis is critical for the health and efficient milk production of dairy cows. During the perinatal period and at peak lactation, milk yield in dairy cows is critically dependent on lactose biosynthesis. Glucose, the primary substrate for lactose synthesis, is predominantly supplied via hepatic gluconeogenesis [28]. In this study, we employed metabolomics to identify potential factors contributing to differences in hepatic gluconeogenesis efficiency between dairy cows and pigs, revealing that bile acids may play a significant role. We further investigated the effects of TDCA, TCA, and GCA in bovine hepatocytes and demonstrated that they predominantly promoted hepatic gluconeogenesis via the TGR5/cAMP/PKA signaling axis, thereby providing precursors for lactose synthesis and supporting milk production in dairy cows (Fig. 7).

While cows (ruminants) and pigs (monogastric animals) exhibit fundamental dietary distinctions that influence hepatic metabolic profiles, species-specific intrinsic biological characteristics constitute key determinants of metabolic regulation, which is the methodological premise of this study. This premise is supported by comparative metabolomic analyses of hepatic bile acids and their downstream metabolites across nine species with divergent dietary habits [29]. Interspecies comparisons of bile acid profiles in humans, pigs, and mice further substantiate this concept [4]. Collectively, these studies establish the critical regulatory role of host-specific factors in shaping bile acid metabolism.

There are already numerous reports highlighting the critical role of bile acids in maintaining glucose and lipid homeostasis [17, 30, 31]. CDCA modulates transcription of key gluconeogenic genes (*PCK*, *G6PC*) via FXR activation, thereby regulating the conversion of non-carbohydrate substrates to glucose [32]. The intestinal TGR5 activation promotes glucagon-like peptide-1 release, stimulating insulin secretion from pancreatic β-cells to maintain systemic glucose balance [33]. Moreover, bile acids supplementation significantly enhances lactational performance in dairy cows during peak lactation [34], suggesting hepatic enrichment of specific BAs may promote gluconeogenesis to support milk synthesis. However, the functional involvement of cow-enriched bile acids (TDCA, TCA and GCA) in hepatic gluconeogenesis remains unexplored. This study therefore addressed the primary objective of determining whether these bile acids regulated hepatic gluconeogenic efficiency in dairy cows.

Our findings demonstrated that TDCA, TCA, and GCA enhanced glucose production in bovine hepatocytes by upregulating expression of gluconeogenic genes (*FBP1*, *PCK1* and *G6PC*). Notably, these BAs exhibited synergistic effects on gluconeogenesis promotion. BAs-mediated regulation of gluconeogenic genes occurred through CREB activation, whereby phosphorylated CREB recruited co-activator CRTC2 to form a transcriptional regulatory complex [9]. CREB phosphorylation is regulated by multiple kinases including PKA, MAPK, AKT, and PKC [9, 35–37]. In the context of gluconeogenesis regulation, the phosphorylation of CREB and its binding to CRTC2 are primarily mediated by PKA [9]. Consistent with this mechanism, we observed increased intracellular cAMP concentration in bovine hepatocytes, triggering PKA activation, subsequent CREB phosphorylation, and enhanced CREB-CRTC2 binding, thereby initiating transcriptional activation.

Bile acid functions typically require receptor mediation. Our receptor profiling revealed FXR non-involvement but identified TGR5 as the critical mediator. BA-induced gluconeogenesis promotion was abolished following TGR5 inhibition or knockdown, confirming the essential role of TGR5 in the BAs-PKA-CREB pathway. While TCA and TDCA are established TGR5 ligands [38, 39], but whether GCA serves as a TGR5 ligand remains unreported, representing a significant gap in knowledge that merits in-depth exploration.

Finally, we validated the in vivo functions of BAs through mouse model. Consistently, the supplementation of BAs in the diet resulted in the enhanced expression of pivotal gluconeogenic genes within mouse livers. Notably, we employed pyruvate tolerance tests and fasting blood glucose measurements to validate the enhancement of gluconeogenic capacity conferred by BAs in mice. Regarding the observed elevation in overall blood glucose levels in SBI-115-treated mice, we compared this phenomenon with the effects of another TGR5 inhibitor, triamterene, on blood glucose regulation. This may represent effect resulting from the complete inhibition of TGR5, characterized by reduced GLP-1 secretion, which subsequently suppresses insulin secretion [40, 41]. However, it is imperative to highlight that the translational potential of BAs as a nutritional strategy to improve milk production efficiency in dairy cows cannot be inferred solely from mouse models. Further investigations involving targeted dietary supplementation in dairy cows are essential to corroborate these findings, thereby bridging the translational gap from preclinical research to practical agricultural applications.

## Conclusions

Based on the differences in gluconeogenesis efficiency between ruminant and monogastric animals, metabolites that potentially regulated gluconeogenesis were investigated in the livers of Holstein dairy cows (ruminant animals) and DLY crossbred pigs (monogastric animals). It was found that TDCA, TCA, CA, and GCA exhibited significant differences, and TDCA, TCA, and GCA alone or in combination (BAs) could promote hepatic gluconeogenesis. By exploring the mechanism of action of BAs, it was revealed that the transcription factor CREB was involved and BAs influenced intracellular cAMP concentration and PKA activation. Finally, considering that the action of BAs required receptor participation, receptor screening identified TGR5 as the mediator of this process. In summary, TDCA, TCA, and GCA enriched in the liver of dairy cows regulated gluconeogenesis via the TGR5/cAMP/PKA/CREB pathway.

## Supplementary Information

Additional file 1: Fig. S1. The composition of metabolites in non-targeted and targeted metabolic profiling of the liver tissue. Fig. S2. The effects of TDCA, TCA, and GCA on FBP2 and PCK2, as well as the influence of CA on the protein and mRNA expression levels of key gluconeogenesis enzymes. Fig. S3. The influence of BAs on the mRNA expression levels of key gluconeogenesis enzymes under FXR-inhibited or TGR5-inhibited conditions. Table S1. qPCR primer sequences.

## Data Availability

The sources of all the materials used in this article have been clearly stated. The datasets supporting the conclusions of this article can be made available on reasonable request to the corresponding author.

## Abbreviations

AKT: Protein kinase B
BAs: Bile acids
BCA: Bicinchoninic acid assay
BSA: Bovine serum albumin
BH: Bovine hepatocyte
CA: Cholic acid
cAMP: Cyclic adenosine monophosphate
CDCA: Chenodeoxycholic acid
CK 18: Cytokeratin 18
CREB: cAMP-response element binding protein
CRTC2: CREB regulated transcription coactivator 2
DLY: Duroc-Landrace-Yorkshire
DMSO: Dimethylsulfoxide
FBP1/2: Fructose 1,6-bisphosphatase
FOXO1: Forkhead box O1
FXR: Farnesoid X receptor
G6PC: Glucose 6-phosphatase
GCA: Glycocholic acid
GPCRs: G protein-coupled receptors
GTT: Glucose tolerance test
GUG: Guggulsterone
HA: Haemagglutination
HCA: Hierarchical clustering analysis
HNF4α: Hepatic nuclear receptor 4α
KEGG: Kyoto encyclopedia of genes and genomes
LC-MS: Liquid chromatography-mass spectrometry
MAPK: Mitogen-activated protein kinas
mTOR: Mammalian target of rapamycin
OPLS-DA: Orthogonal partial least squares discriminant analysis
PBS: Phosphate buffer saline
PCA: Principal component analysis
PCC: Pearson correlation coefficient
PCK1/2: Phosphoenolpyruvate carboxykinase
PEI: Polyethylenimine
PGC-lα: Peroxisome proliferator-activated receptor-y coactivator-1α
PKA: Protein kinase A
PKC: Protein kinase C
PMSF: Phenylmethylsulfonyl fluoride
PTT: Pyruvate tolerance test
qPCR: Quantitative PCR
RIPA: Radioimmunoprecipitation assay buffer
RLU: Relative light units
RT: Reverse transcription
SPF: Specific pathogen-free
TCA: Taurocholic acid
TDCA: Taurodeoxycholic acid
TGR5: Takeda G protein-coupled receptor 5
VIP: Variable importance in projection
