# Supplementary material for: Taurodeoxycholic, taurocholic, and glycocholic acids promote hepatic gluconeogenesis via TGR5 in dairy cows
Source: J Anim Sci Biotechnol. 2025 Oct 30;16:142. doi: 10.1186/s40104-025-01275-w (PMC12574133; doi:10.1186/s40104-025-01275-w)
Supplement: Supplementary file 1 — Additional file 1: Fig. S1. The composition of metabolites in non-targeted and targeted metabolic profiling of the liver tissue. Fig. S2. The effects of TDCA, TCA, and GCA on FBP2 and PCK2, as well as the influence of CA on the protein and mRNA expression levels of key gluconeogenesis enzymes. Fig. S3. The influence of BAs on the mRNA expression levels of key gluconeogenesis enzymes under FXR-inhibited or TGR5-inhibited conditions. Table S1. qPCR primer sequences. [file 40104_2025_1275_MOESM1_ESM.pdf]

Supplementary Information

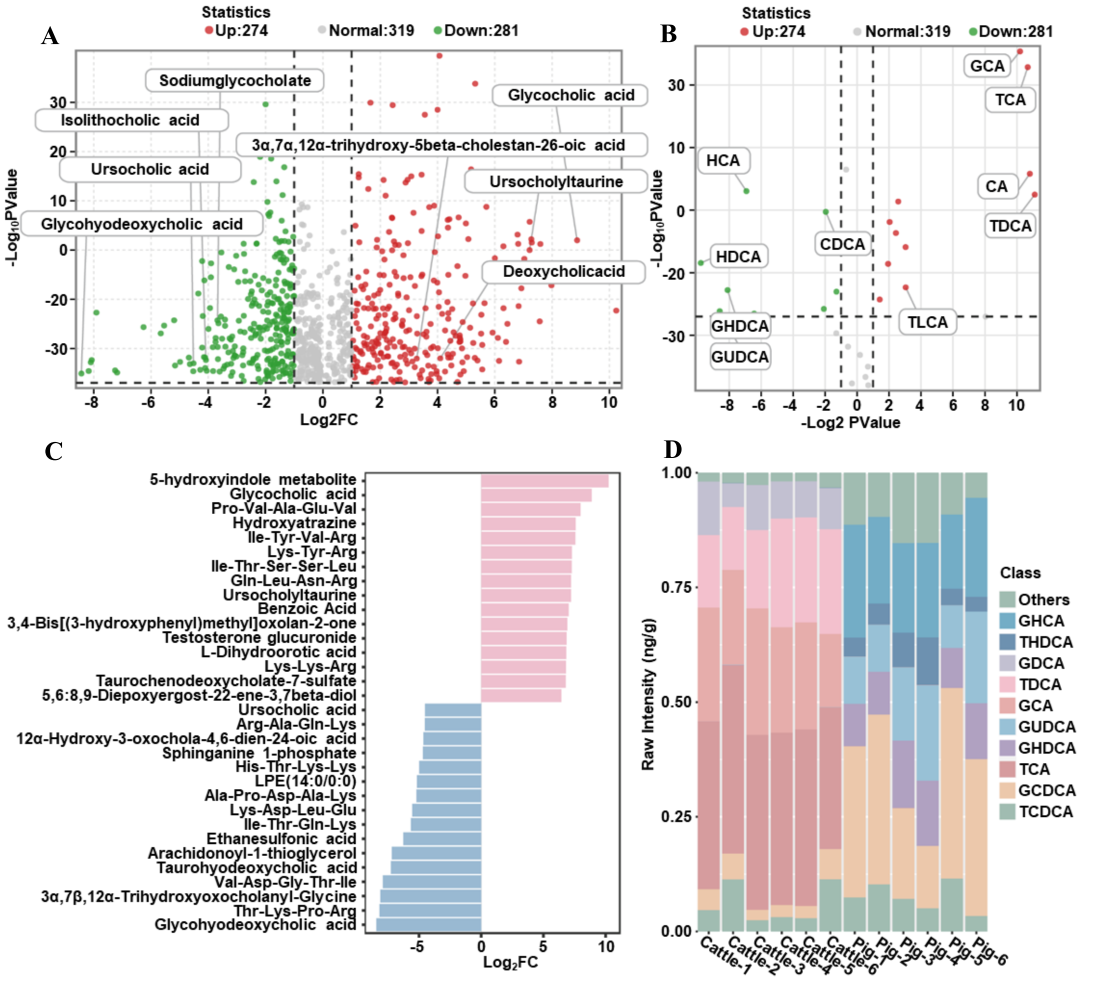

Figure S1. The composition of metabolites in non-targeted and targeted metabolic profiling of the liver tissue.

A Volcano plot of differentially expressed metabolites identified by non-targeted metabolomics in the liver tissues of Holstein cows and Tri-Gen hybrid pigs. **B** Bar chart showing the fold changes of metabolites identified by non-targeted metabolomics in liver tissues. **C** Volcano plot of differentially expressed bile acids identified by targeted metabolomics in liver tissues. **D** Composition ratios of bile acids in individual liver samples.

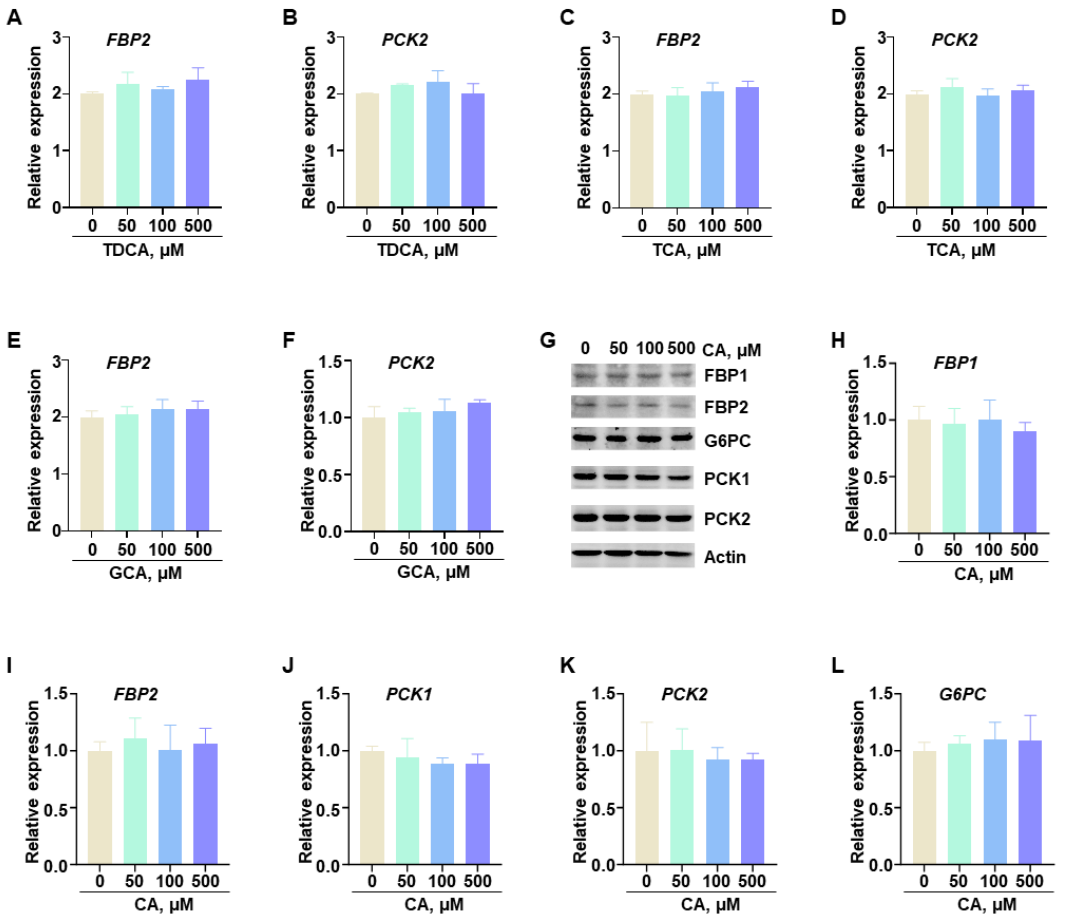

Figure S2. The effects of TDCA, TCA, and GCA on FBP2 and PCK2, as well as the influence of CA on the protein and mRNA expression levels of key gluconeogenesis enzymes.

A-B The mRNA expression levels of FBP2 and PCK2 after BH stimulation by TDCA (n=3). **C-D** The mRNA expression levels of FBP2 and PCK2 after BH stimulation by TCA (n=3). **E-F** The mRNA expression levels of FBP2 and PCK2 after BH stimulation by GCA (n=3). **G** Protein expression levels of gluconeogenesis rate-limiting enzymes after BH stimulation by CA. **H-L** mRNA expression levels of gluconeogenesis rate-limiting enzymes after BH stimulation by CA (n=3). All data were represented as mean ± SEM. $*P < 0.05$, $**P < 0.01$ and $***P < 0.001$ compared to the CON group.

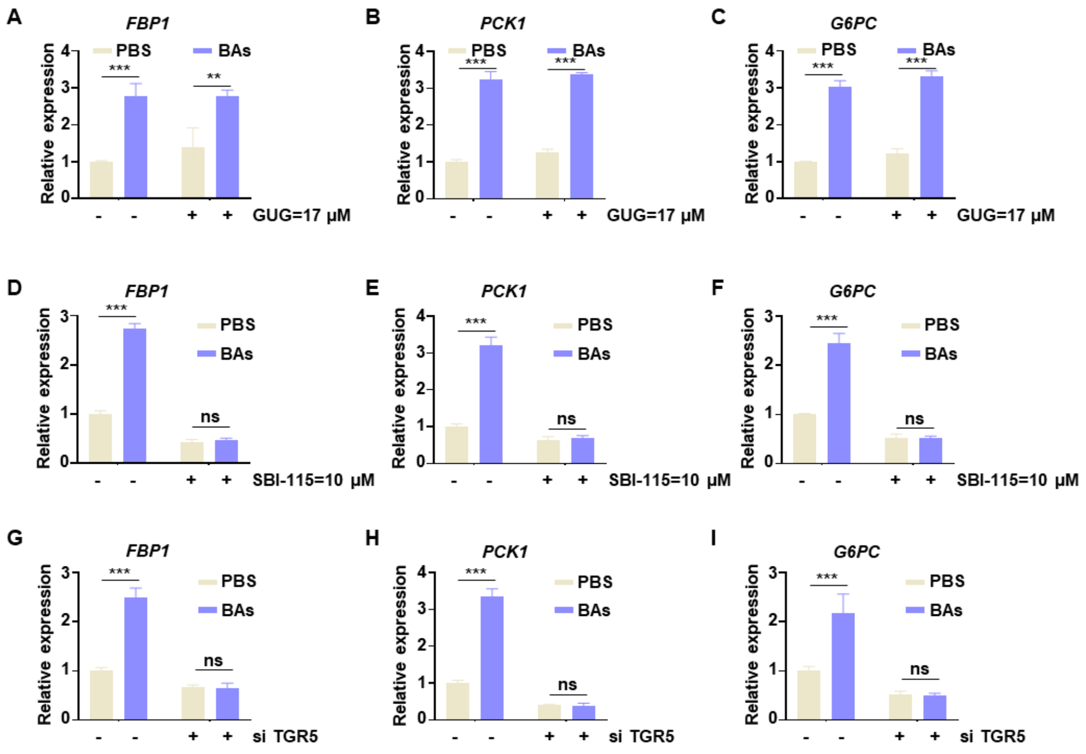

Figure S3. The influence of BAs on the mRNA expression levels of key gluconeogenesis enzymes under FXR-inhibited or TGR5-inhibited conditions.

A-C The mRNA expression levels of FBP1, PCK1, and G6PC in BH after adding BAs with FXR inhibitors (GUG) (n=3). **D-F** The mRNA expression levels of FBP1, PCK1, and G6PC in BH after adding BAs with TGR5 inhibitors (SBI-115) (n=3). **G-I** The mRNA expression levels of FBP1, PCK1, and G6PC in BH after adding BAs with TGR5 knockdown (n=3). All data were represented as mean ± SEM. *$P < 0.05$, **$P < 0.01$ and ***$P < 0.001$ compared to the CON group.

Table S1: qPCR primer sequences

Gene	Forward primer sequence (5’→3’)	Reverse primer sequence (5’→3’)
FBP1	TCCTGCCCTCACCGAGTATG	TCATACAGTAGTCTCAGCTTTCCA
PCK1	GACGGCCTCAACTACTCAGC	AGTGAGAGCCAACCAGCAGT
G6PC	ACTCCTCTGGGTAGCTGTGAT	ACATGACATTCAAGCACCGAAAT
TGR5	GGACAACTCCCTGACACTCG	GGCATGCATGACTGTAGGT
β-Actin	AAGGACCTCTACGCCAACACG	TTTGCGGTGGACGATGGAG
